# Exploring the mechanisms underlying excitation/inhibition imbalance in human iPSC-derived models of ASD

**DOI:** 10.1186/s13229-020-00339-0

**Published:** 2020-05-11

**Authors:** Lorenza Culotta, Peter Penzes

**Affiliations:** 1 Department of Physiology, Northwestern University Feinberg School of Medicine, Chicago, IL USA; 2 Department of Psychiatry and Behavioral Sciences, Northwestern University Feinberg School of Medicine, Chicago, IL USA; 3 Center for Autism and Neurodevelopment, Northwestern University, Chicago, IL USA

**Keywords:** Autism spectrum disorder, Induced pluripotent stem cell, Excitation/inhibition balance

## Abstract

Autism spectrum disorder (ASD) is a range of neurodevelopmental disorders characterized by impaired social interaction and communication, and repetitive or restricted behaviors. ASD subjects exhibit complex genetic and clinical heterogeneity, thus hindering the discovery of pathophysiological mechanisms. Considering that several ASD-risk genes encode proteins involved in the regulation of synaptic plasticity, neuronal excitability, and neuronal connectivity, one hypothesis that has emerged is that ASD arises from a disruption of the neuronal network activity due to perturbation of the synaptic excitation and inhibition (E/I) balance. The development of induced pluripotent stem cell (iPSC) technology and recent advances in neuronal differentiation techniques provide a unique opportunity to model complex neuronal connectivity and to test the E/I hypothesis of ASD in human-based models. Here, we aim to review the latest advances in studying the different cellular and molecular mechanisms contributing to E/I balance using iPSC-based in vitro models of ASD.

## Background

Autism spectrum disorder (ASD) represents a spectrum of early-onset neurodevelopmental disorders characterized by persistent deficits in social interaction and communication, as well as repetitive patterns of behavior and restricted interests or activities (Diagnostic and Statistical Manual of Mental Disorders [DSM-5]). Current population prevalence of ASD is estimated at ∼ 1.5% in developed countries around the world and, to date, there are no effective cures [1, 2]. ASD patients display considerable phenotypic heterogeneity and they often present comorbid neurological and mental conditions, such as epilepsy, intellectual disability (ID), obsessive-compulsive disorder (OCD), and attention-deficit hyperactivity disorder (ADHD) [3].

ASD can be classified into syndromic and non-syndromic forms [4–7]. Syndromic ASD accounts for a small percentage of total ASD cases; it typically occurs with a clinical presentation in association with secondary phenotypes and/or dysmorphic features [6]. Most of the syndromic forms of ASD have a known genetic cause, often involving chromosomal abnormalities or mutations in a single gene. On the other hand, non-syndromic ASD accounts for the vast majority of ASD cases and occurs without additional symptoms. In contrast to the syndromic ASD, most of the non-syndromic forms of ASD have unknown genetic etiology [5]. Even though the genetics underlying ASD is complex, ASD is primarily considered a genetic disorder, with family and twins studies suggesting the heritability of ASD to be higher than 80% [8–12]. Genetic studies have identified several autism-susceptibility (or risk) genes (e.g., SHANK- and NRXN-family genes) and copy number variation (CNV) loci (e.g., 16p11.2 deletion and 15q11-q13 duplication), facilitating the molecular diagnosis of ASD cases. Many of the identified ASD risk genes are key regulators of synaptic plasticity, with their gene products involved in modulating synaptic strength or density, cell adhesion, chromatin remodeling, transcription, cytoskeleton dynamics and, ultimately, neuronal connectivity [13–15]. In addition to genetic risk factors, several studies suggest that environmental factors may contribute to ASD risk, supporting the hypothesis that ASD could result from the effects of diverse biological and/or psychological factors, like genetic factors, environmental factors, and the interplay between genetic background and environmental factors [16–18]. The environmental factors could be prenatal, perinatal, and postnatal, and some examples include in utero exposure to medications, such as thalidomide and valproate [19, 20], parental age of birth, and gestational complications such as diabetes and bleeding [11, 21]. The speculated existence of interactions between genetic factors and environmental factors suggests that individuals with ASD may react differently to the same environmental stimulus: indeed, studies of animal models have shown that environmental factor, such as hypoxia, oxidative stress, and maternal immune activation, may increase autism risk by interacting with genetic defects in synaptic function [22–24].

One of the proposed etiological mechanisms of ASD is the disruption of the balance between excitation and inhibition (E/I balance) in key cortical and subcortical neuronal circuits [25–30]. E/I balance is a crucial player in the normal development and function of the brain, and different homeostatic and developmental processes appear to be involved in maintaining E/I balance at the level of single cells and large-scale neuronal circuits [26]. At the single neuron level, the balance between excitatory and inhibitory synaptic inputs is critical for information processing, and therefore is highly regulated and structurally organized to allow spatially precise E/I balance across dendritic segments [31, 32]. At the network level, E/I balance is usually considered as a stable global level of activity within a particular circuit, being the balance of excitation and inhibition important for optimal tuning of the circuits to respond to salient inputs [33, 34]. To date, several studies have described different neurobiological mechanisms contributing to the establishment and rigorous regulation of the E/I balance. Specific factors include intrinsic neuronal excitability, synaptic transmission, and homeostatic synaptic plasticity; at the circuit level, key contributors to E/I balance are the complex interplay between glutamatergic excitatory neurons and GABAergic inhibitory neurons and the development of excitatory and inhibitory synapse, as well as the overall neuronal network excitability [15, 35–37].

A growing body of clinical neuroimaging literature supports the role of E/I imbalance as an etiology of ASD [28, 29]. Gamma-band electrophysiological activity (30–100 Hz) is considered to be a functional readout of E/I balance within local neural circuits [38]. Studies of gamma-based activity via magnetoencephalography (MEG) and electroencephalography (EEG) have identified alterations in gamma-band activity in ASD patients, especially in relation to auditory-related gamma-band activity; however, despite providing indirect evidence for E/I imbalance in ASD, gamma-based activity fundamentally remains a proxy measure [39, 40]. Magnetic resonance spectroscopy (MRS) has allowed for the direct non-invasive, in vivo estimation of the principal excitatory and inhibitory neurotransmitters, glutamate, and gamma-aminobutyric acid (GABA), and alterations of neurotransmitter levels have been reported within different cortical structures in ASD patients [41–44]. Disruptions in excitation-inhibition ratio have been suggested to correlate with the severity of core ASD symptoms, thus implying that the abnormality in E/I balance is clinically relevant. In particular, glutamate levels in the striatum have been found to negatively correlate to severity of social impairment [42], while a positive correlation has been reported between cortical excitation-inhibition ratio and the severity of autistic phenotypes in non-ASD psychiatric subjects [29]. However, these reports are widely inconsistent, possibly due to the different methodologies employed as well as the heterogeneity of subjects between studies, and it is still unknown whether any perturbation in neurotransmitter level reflects a neurotransmission phenotype, rather than a metabolic phenotype [28].

The advent of the induced pluripotent stem cell (iPSC) technology has allowed the generation of personalized human neurons, thus representing a new avenue for the modeling of neurological disorders [45–47]. This model has been made possible through the reprogramming of patient-derived somatic cells, e.g., fibroblasts [46], peripheral blood mononuclear cells [48], or urine cells [49], into pluripotent stem cells via the overexpression of a set of pluripotency-associated transcription factors, Oct4, Sox2, Klf4, and cMyc, named the “Yamanaka factors” [46]. iPSCs have a gene expression profile and pluripotency similar to human embryonic stem cells (hESCs), but with the advantage of easy accessibility and the avoidance of ethical and religious concerns regarding the use of human embryos for research purposes [50, 51]. Given their potential to differentiate into virtually any cell type under appropriate culture conditions, iPSCs could represent a valuable source of disease-relevant cells that were previously inaccessible, such as cardiomyocytes and neurons. As iPSCs can be differentiated into a range of neuronal lineages, iPSC-based disease modeling has greatly contributed to our understanding of neurological diseases, serving as a precious complement to animal models for analyzing neuronal development and the consequences of its dysfunctions [46]. This provides an incredibly strong model of disease by recapitulating the entirety of the complex genetic factors that result in patient diagnosis. Therefore, the iPSC approach provides an invaluable tool for investigating the cellular and molecular mechanisms underpinning complex genetic diseases, such as ASD and its related disorders, which include Rett syndrome (RTT), Timothy syndrome (TS), fragile X syndrome (FXS), and Phelan-McDermid syndrome (PMS) [52].

**Fig. 1:**
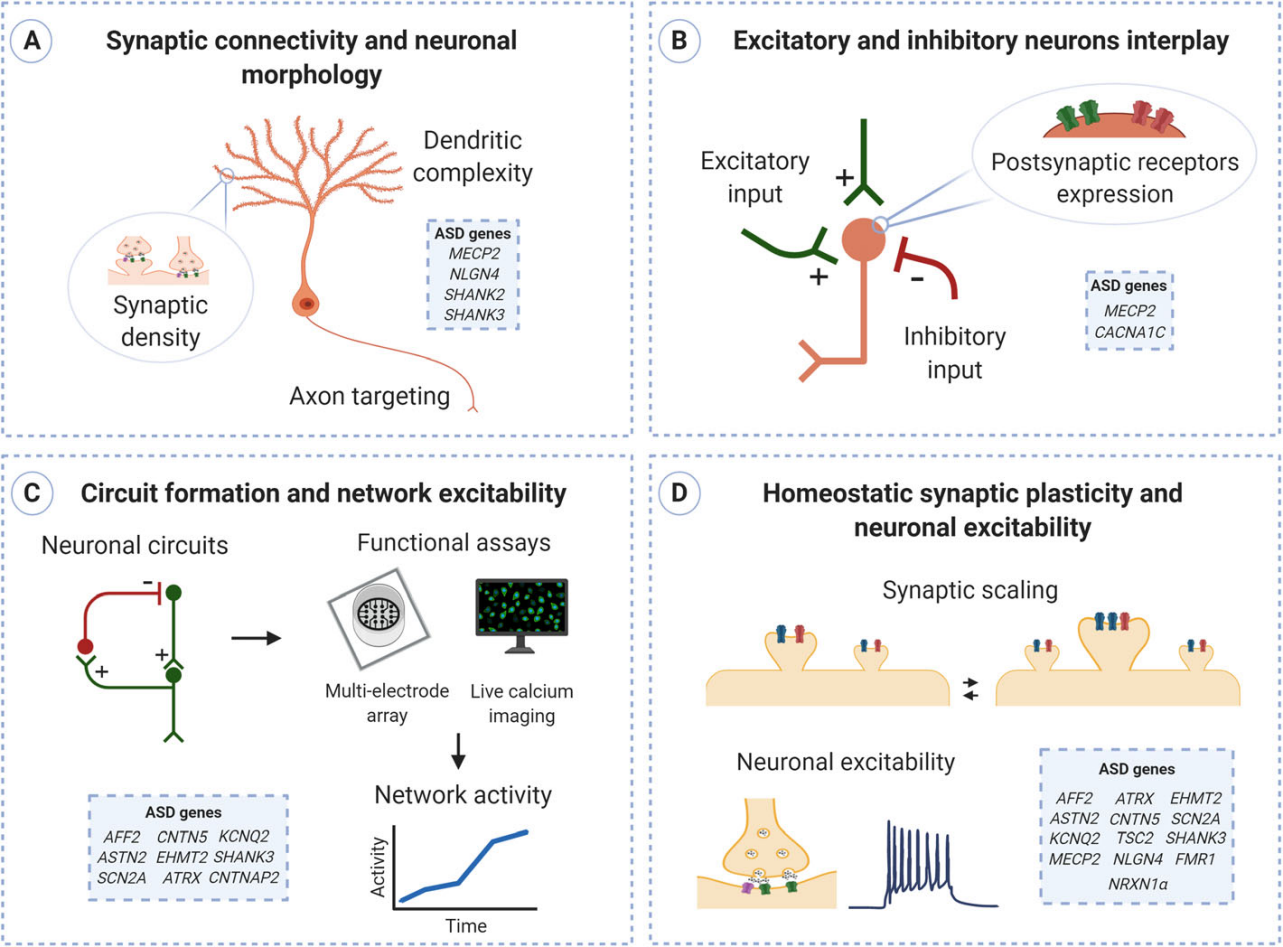
Neurobiological mechanisms contributing to E/I balance. Schematic illustration of key mechanisms involved in establishing and regulating the balance between excitation and inhibition, highlighting the mechanisms and the ASD-related genes discussed in this review. In the cited reports, the iPSC technology has been exploited to develop human-based platforms in which to investigate the contribution of ASD-related genes to the different processes underlying E/I balance. **a** Establishment and maintenance of neuronal connectivity and E/I balance require all the salient features of neuronal morphology: the existence of branching dendrites and axons and the presence of neuronal synapses. Alterations in one or more of these features have been reported in several ASD models. **b** Excitatory and inhibitory neuron interplay results from the excitatory and inhibitory inputs converging on a neuron, as well as from the level of expression of postsynaptic glutamatergic (green) and GABAergic (red) receptors. **c** The application of technologies such as multi-electrode arrays and live calcium imaging has facilitated real-time, multi-point measurement of the activity of iPSC-derived neurons and allowed investigating developmental modifications of synaptic connectivity and network activity. **d**. Synaptic scaling is a form of homeostatic plasticity that operates to modify the global synaptic input (excitability) of a neuron in response to changes in circuit activity. One of the main mechanisms of synaptic scaling is the modification of the density and/or the size of synapses, and multiple electrophysiological techniques are used to analyze synaptic plasticity and neuronal excitability, including miniature excitatory/inhibitory postsynaptic currents

In this review, we will focus on human iPSC-derived in vitro models of ASD and summarize the most recent studies in which the iPSC technology has been exploited to investigate the molecular bases of E/I imbalance and to gain further insight into the etiological mechanisms of ASD (Table 1). Being E/I imbalance the focus of this review and being ASD a neurodevelopmental disorder, we decided to include in this review only reports on stem cell-derived neuron in which functional alterations that could impact E/I balance have been reported (Fig. 1). Moreover, given the growing number of ASD risk genes [81], we decided to include only risk genes belonging to SFARI categories 1 and 2, thus excluding genes without strong evidence supporting the gene’s relevance to ASD risk.

**Table 1:** iPSC-based models of ASD discussed in this review

Gene	Model type	Phenotype observed	Reference
*ATRX*, *AFF2*, *KCNQ2*, *SCN2A*, and *ASTN2*	Homozygous deletion	Reduced synaptic activity	[53]
*CACNA1C*	ASD-related mutations	Disrupted interneurons migration	[54]
*CNTN5* and *EHMT2*^+^	Heterozygous deletion	Hyperexcitability.	[55]
*CNTNAP2*	Heterozygous deletion	Increased neuronal network activity	[56]
*FMR1*	Heterozygous deletion	Impaired retinoic acid (RA)-dependent homeostatic synaptic plasticity	[57]
*MECP2*	Heterozygous deletion or duplication	Altered synaptic density, altered calcium signaling; altered neuronal firing rate and synchronization; delayed GABA switch	[58–61]
*NLGN4*	Gene overexpression and ASD-related mutations	Increased excitatory synapse density, altered synaptic strength	[62, 63]
*NRXN1α*	Homozygous and heterozygous deletion, ASD-related mutations	Impaired synaptic strength, altered synaptic calcium signaling	[64–66]
*SHANK2*	Heterozygous deletion and ASD-related mutations	Hyperconnectivity, enhanced branching complexity, increased synapse density	[67]
*SHANK3*	Heterozygous deletion and ASD-related mutations	Hypoconnectivity, reduced synaptogenesis, and dendritic arborization; impaired neuronal excitability and excitatory synaptic transmission; impaired HCN channels	[68–74]
*TSC1/2*	Homozygous and heterozygous deletion	Altered neuronal excitability and activity, altered synchrony (cortical neurons); hypoexcitability (cerebellar Purkinje cells)	[75–78]
*Other ASD models*		Aberrant neuronal maturation, altered neuronal differentiation and synaptic formation	[79, 80]

## Main text

### Neuronal excitability and excitatory synaptic transmission

In vitro neuronal cultures provide insight into neuronal transmission and network interactions since they retain many of the properties of in vivo networks, such as functional synaptic connectivity and complex patterns of neuronal activity [82, 83]. Considering that defective neuronal networks, as well as reduced functional connectivity, have been widely observed in ASD patients [84], iPSC-derived neuronal cultures provide a valuable method for modeling neuronal activity, allowing the investigation of the intrinsic neuronal excitability, formation of synaptic connections, and overall network activity.

The use of multi-electrode array (MEA) technology has facilitated non-invasive, real-time, multi-point measurement of the activity of cultured neurons and allowed investigating developmental modifications of synaptic connectivity and network activity [85–87]. Therefore, the MEA system is acknowledged as a suitable method for evaluating the plasticity of iPSC-derived neuronal networks and for investigating the molecular basis of the E/I imbalance in ASD.

Deneault et al. utilized MEA recordings to evaluate the neuronal network properties in iPSC-derived neurons and to assess the impact of ASD genetic variants on synaptic and intrinsic electrophysiological properties [53, 55]. Multiple intriguing associations were observed between the genetic variants and the neuronal phenotypes analyzed: one of the most robust findings was a marked hyperexcitability in glutamatergic neurons lacking one copy of *CNTN5* or *EHMT2* [55], as well as a reduced synaptic activity in *ATRX-*, *AFF2-*, *KCNQ2-*, *SCN2A-*, and *ASTN2*-null neurons [53]. Together, these data indicate that ASD-risk genes belonging to different gene ontologies can produce a similar electrophysiological phenotype and reveal common functional phenotypes, such as altered functional connectivity, which could ultimately result in altered excitation/inhibition balance.

#### *SHANK* family

A growing body of literature pinpoints inadequate synaptogenesis and perturbed excitatory synaptic plasticity as key players in the development of dysfunctional neuronal networks and, ultimately, in the E/I imbalance [15, 88–90]. A valid example is represented by mutations in the *SHANK* family genes, which encode major scaffolding proteins in the excitatory postsynaptic density [91–94]. Loss-of-function mutations in the *SHANK2* gene have been associated with various neuropsychiatric disorders, such as ASD, intellectual disability, and schizophrenia [95]. In mouse models and primary cultures of neurons, *Shank2* has been demonstrated to serve an important role in early neuronal development, neuronal dendrite branching, and basal synaptic transmission [96–99]. In line with these findings, loss-of-function mutations in *SHANK2* have been associated with altered connectivity in neurons derived from ASD-affected individuals, as well as CRISPR-Cas9-edited homozygous *SHANK2* knockout (KO) neurons [67]. *SHANK2* mutant neurons displayed enhanced dendrite branching complexity alongside increased synapse number and spontaneous excitatory postsynaptic currents, thus resulting in strengthened functional neuronal connectivity. Remarkably, the reversal of this phenotype in homozygous KO neurons via engineering correction of the mutation provides a robust demonstration of the causal role of *SHANK2* mutation in determining the functional hyperconnectivity observed.

This phenotype is distinct from the lowered connectivity associated with mutations in *SHANK3* [100–102]. *SHANK3* gene mutations are strongly linked to ASD, and *SHANK3* haploinsufficiency is believed to be the major contributing factor to the 22q13.3 deletion syndrome, also known as Phelan-McDermid syndrome (PMS) [103]. Multiple iPSC-based studies sought to assess the functional significance of *SHANK3* mutations, using conditional *SHANK3*-mutant neurons and/or PMS and ASD patient-derived neurons [68–73]. Confirming the crucial role of SHANK3 protein in the regulation of synaptic plasticity, *SHANK3* haploinsufficiency resulted in the disruption of synaptogenesis and dendritic complexity, which are critical to ensure proper neuronal connectivity. Indeed, *SHANK3-*deficient iPSC-derived neurons exhibited impaired dendritic arborization, synaptic density, and intrinsic excitability along with defective excitatory synaptic transmission. Interestingly, Yi et al. have proposed a new role for SHANK3, in addition to having a specifically postsynaptic function [71]. In their work, the authors reported a severe impairment in hyperpolarization-activated cation currents (I_h_) in *SHANK3*-deficient hESC-derived neurons. Considering that I_h_ currents are mediated by hyperpolarization-activated cyclic nucleotide-gated (HCN) channels, the authors speculated that SHANK3 might scaffold HCN channels during neurodevelopment [71]. Notably, HCN-channel mutations are associated with dysfunctional neuronal firing and therefore have been linked to neurological disorders such as epilepsy and impaired learning [104].

Altogether, these findings highlight the relevance of *SHANK* genes’ dosage to neuronal intrinsic excitability and excitatory synaptic transmission, whose disruptions would directly affect E/I balance.

#### Adhesion molecules

Defective synaptic transmission has been observed in human neurons carrying mutations in several ASD-associated genes encoding synaptic adhesion molecules. *NLGN4* is a member of the neuroligin family and encodes the postsynaptic adhesion molecule Neuroligin-4. Deletions at Xp22.3 encompassing *NLGN4* as well as de novo mutations in the *NLGN4* gene have been reported in several ASD patients [105]. Neuroligin-4 has been suggested to be an essential element in the regulation of excitatory synaptic transmission. In fact, stem cell-derived neurons overexpressing *NLGN4* or carrying ASD-related *NLGN4* mutations have been reported to have increased excitatory synapse density and altered synaptic strength, thus showing a deleterious effect of *NLGN4* dysregulation on synaptic transmission and E/I balance [62, 63, 106].

Impaired synaptic function has been also reported in human neurons carrying *NRXN1α* mutations. *NRXN1α* encodes the presynaptic cell-adhesion molecule Neurexin-1; moreover, mutations and copy number variations of *NRXN1α* have been associated with ASD and schizophrenia [107]. iPSC technology has been widely exploited to investigate the functional significance of disease-associated mutation of *NRXN1α* in synaptic transmission. Evidence showed that mutations of *NRXN1α* severely impaired synaptic transmission by decreasing neurotransmitter release [64]. Controversial results have been obtained when evaluating calcium signaling activity in *NRXN1α-*mutant human neurons: in fact, biallelic deletion of *NRXN1α* has been associated to decreased calcium dynamics, suggesting decreased intrinsic activity [65], while neurons carrying NRXN1α^+/−^ deletions display increased calcium activity [66]. Although seemingly contradictory, these results show the profound impact of *NRXN1α* loss of function on synaptic excitation, further supporting the E/I imbalance hypothesis in ASD.

Altered neuronal network activity has also been associated with heterozygous deletion of *CNTNAP2*. Contactin-associated protein-like 2 (CNTNAP2) is a member of the neurexin family, and gene mutations and common variation in the *CNTNAP2* gene have been associated with neurodevelopmental disorders, including ASD [108]. Consistent with reports of disrupted cortical neuronal activity and increased propensity for seizure-like activity in CNTNAP2-null mice [109–112], induced glutamatergic neurons derived from carriers of heterozygous intragenic CNTNAP2 deletions displayed increased neuronal network activity, reflected by the increase in spontaneous spiking activity recorded via MEA, consistent with previous reports [56].

#### *TSC1/2*

Tuberous sclerosis complex (TSC) provides an example of ASD-associated neuronal excitability abnormalities detected in cortical and non-cortical areas. TSC is a severe multisystem disorder associated with neurologic symptoms including autism, epilepsy, and cognitive disability [113]. TSC is caused by heterozygous loss-of-function mutations in either the *TSC1* or *TSC2* gene, which are key inhibitory components of the mTORC1 pathway, a regulator of cell development and synaptic homeostasis. Given the implication of enhanced activity of the mTORC1 pathway with ASD, and the high prevalence of ASD in TSC patients [114], several groups have generated iPSC-derived neurons carrying *TSC1/2* mutations to investigate the effects of altered TSC-mTORC1 pathway on neuronal development and synaptic physiology. Surprisingly, functional analyses of *TSC*-mutated neurons led to controversial results. Costa et al. reported reduced excitability in *TSC2*^*−/−*^ stem cell-derived neurons and a milder phenotype in *TSC2*^*+/−*^ neurons, albeit significantly disrupted [75]. On the contrary, Nadadhur et al. observed increased spontaneous calcium event frequency as well as neuronal hyperactivity in TSC patient-derived neuronal networks [76]. Winden et al. also confirmed increased neuronal activity in *TSC2*^*−/−*^ neurons using multi-electrode arrays, and observed increased neuronal synchrony in both *TSC2*^*+/−*^ and *TSC2*^*−/−*^ neurons. Despite opposing effects in activity, these results suggest an abnormality in neuronal connectivity, which may be affected in alternate ways depending on the genetic background of patients [77]. Interestingly, mTORC1 inhibition via rapamycin treatment corrected the excitability deficits both in the case of increased and decreased neuronal activity [75, 77, 113]. These results confirm that deregulated mTORC1 pathway can result in varying phenotypes that, despite seemingly opposing each other, in fact highlight that *TSC2* loss of function differentially affects neuronal excitability and, ultimately, E/I regulation.

A high prevalence of ASD in TSC individuals has been associated with cerebellar abnormalities, and dysregulation of the cerebellar cortical development has been suggested as a possible mechanism involved in the development of ASD in TSC patients [115]. In this light, cerebellar Purkinje cells (PC) were derived from TSC patients with ASD, with the aim to characterize the molecular mechanisms of cerebellar dysfunction in ASD and TSC: *TSC2*-deficient PC have been found to display severe hypoexcitability, reflected by decreased resting membrane potential and lowered spontaneous activity [116].

### Homeostatic synaptic plasticity

Homeostatic synaptic plasticity is a form of neuronal plasticity that operates to adjust the strength of synapses in response to changes in neural activity, thereby refining neuronal connectivity during development and contributing to network stability [117, 118]. Multiple ASD-associated genes are predicted to influence homeostatic plasticity mechanisms at different levels, as they encode proteins involved in chromatin remodeling, protein synthesis, actin cytoskeleton dynamics, or synaptic transmission [15].

#### *FMR1*

Recent reports indicate that deficits in homeostatic synaptic plasticity might be detrimental for network stability and contribute to cognitive impairment associated with fragile X syndrome (FXS), the most common genetic form of mental retardation with autistic-like behaviors [119]. FXS is caused by the absence of fragile X mental retardation protein (FMRP), an RNA-binding protein involved in dendritic protein synthesis, encoded by the *FMR1* gene. Using FXS patient-derived and conditional *FMR1* knockout (KO) human embryonic stem (hES) cell lines, Zhang et al. demonstrated that FMRP has an essential role in retinoic acid (RA)-dependent homeostatic synaptic plasticity [57]. Synaptic RA signaling is a key component in the regulation of synaptic strength, and RA is necessary to induce local translation and synaptic scaling [120]. Loss of FMRP expression impaired RA-induced synaptic potentiation, blocking the normal increase in excitatory postsynaptic current (EPSC) amplitude following chronic synaptic silencing. These results demonstrate that FMRP-driven local translation is required postsynaptically for synaptic scaling mediated by RA.

#### *MECP2*

*MECP2* is an example of a relevant protein involved in synaptic plasticity, playing an important role in chromatin remodeling. Females with loss-of-function mutations in the X-linked *MECP2* gene display the progressive neurological disorder Rett syndrome (RTT) [121], whereas boys or girls with duplication of *MECP2* develop ASD and ID [122]. It has been demonstrated that MeCP2 plays an essential and cell-autonomous role in homeostatic synaptic plasticity, as neurons lacking MeCP2 failed to trigger synaptic plasticity and to scale synaptic strength bidirectionally [123, 124]. Neurons generated from RTT patient iPSCs have been widely exploited as a model to recapitulate the early stages of this human neurodevelopmental disease. Marchetto et al. have demonstrated the utility of iPSCs to investigate the functional consequences of *MECP2* loss of function. Neurons generated from fibroblasts of RTT patients carrying different *MECP2* mutations exhibited several morphological alterations, including a reduced number of dendritic spines and synapses, and smaller soma size. Moreover, altered intracellular calcium signaling and electrophysiological defects were detected in RTT-derived neuronal networks [58]. Li et al. observed similar synaptic deficits using an isogenic stem cell model of RTT: they reported a significant reduction of action potential rates when recording synchronized events from iPSC-derived neuronal networks [59]. The importance of correct *MECP2* dosage for synaptic plasticity and homeostasis was also investigated in human neurons carrying *MECP2* duplications. Increased glutamatergic synapse number and altered dendritic morphology were observed in *MECP2* duplication neurons, as well as enhanced neuronal network synchronization [60]. These findings confirm a pivotal role of MeCP2 protein dosage in dendritic plasticity and synaptic homeostasis and, ultimately, in neuronal connectivity and electrophysiology.

### Neuron differentiation, maturation, and circuit formation

#### Developmental time points

Many of the genes identified as ASD-risk genes are involved at several time points and in different neural cell types during human cortical development. A commonly used strategy to address this issue is to follow iPSCs during their early neuronal development to examine when and how the earliest ASD-related phenotypes and molecular abnormalities arise. Schafer et al. performed time-series transcriptome and cellular phenotype analyses in ASD neural stem cells (NSCs) and identified the NSC stage as a critical developmental period in which dysregulation of specific transcriptional networks arise. The alterations reported in ASD NSCs were a causal factor in later aberrant neuronal maturation of cortical neurons [79]: notably, skipping the NSC stage by direct conversion of iPSC to neurons prevented neuronal ASD phenotypes from manifesting. A similar approach has been used to explore the role of *SHANK3* in neuronal development. In a recently published work, Huang et al. performed morphological and functional analyses at different time points during neuronal development in iPSC-derived neurons following *SHANK3* knockdown [74]. *SHANK3* depletion resulted in reduced growth cone area, neuronal soma size, and neurite complexity at different developmental time points, together with impaired excitatory and inhibitory synaptic transmission, thus confirming in a human-based model the pivotal role of SHANK3 in early stages of neuronal development, in addition to its well characterized role in mature neuronal function.

#### GABA functional switch

Given that the complex interplay between glutamatergic excitatory neurons and GABAergic inhibitory neurons is vital to achieving proper cortical neural activity, a considerable effort has been made in the stem cell field to generate neuronal networks with balanced excitatory-inhibitory activities, facilitating studies of disease mechanisms involving E/I imbalance [125, 126]. In this context, the GABA functional switch from excitation to inhibition is critical for the efficacy of GABAergic function. KCC2, a membrane K^+^-Cl^−^ cotransporter and one of the major players in defining the transmembrane Cl^−^ gradient, is a critical downstream gene target of MeCP2. Significant KCC2 deficits have been reported in a mouse model of Rett syndrome, alterations that were rescued by treatment with recombinant human insulin-like growth factor-1 [127]. Altered expression of KCC2 has been also observed in iPSC-derived neurons from patients with Rett syndrome [61]. In fact, the lack of MeCP2 in human Rett neurons led to deficient KCC2 expression, and therefore a delayed GABA function switch. As proper inhibitory input is crucial to the development of normal neuronal circuitry, this novel pathway provides insight into the potential mechanisms of Rett syndrome.

#### Brain organoids

The advent of 3D cultures, the most recent stem cell-based model for investigating human brain development and disorders, has provided particular strength to the generation of informative neuronal networks [128, 129]. 3D neuronal cultures, also known as organoids or spheroids, have the potential to recapitulate the structural and functional complexity of the human brain with greater complexity than 2D models. One of the major advantages of the 3D technology is the diversity of cell types present in the organoids, including, but not restricted to, glutamatergic neurons, GABAergic neurons, astrocytes, and oligodendrocytes [130, 131]. Consequently, organoids represent an invaluable and versatile tool to investigate neuronal differentiation and neural circuit formation, essential features underlying network-level E/I balance. For instance, Mariani et al. demonstrated that telencephalic organoids derived from ASD patients present altered neuronal differentiation and synaptic formation, alongside enhanced GABAergic differentiation, which together resulted in an E/I imbalance [80]. Notably, the overproduction of GABAergic neurons was ascribed to overexpression of the transcription factor FOXG1: taken together with the alterations linked to MeCP2, these results reinforce the importance of proper gene expression in ASD.

iPSC-derived spheroids have allowed the assembly of the different neuronal circuits to be modeled, in a manner similar to that which occurs in human cerebral cortex formation [132]. Therefore, they have provided a precious tool to investigate the molecular processes that can lead to an imbalance of cortical excitation and inhibition. Timothy syndrome (TS) is a severe ASD-related neurodevelopmental disorder caused by mutations in the *CACNA1C* gene, encoding the CaV1.2 calcium channel [133]. In a model of TS, neural spheroids were generated to recapitulate the assembling of circuits involving both glutamatergic neurons and GABAergic interneurons and exploited to investigate migration of interneurons and their functional integration into human-derived forebrain spheroids [54]. TS-derived interneurons showed inefficient migration, resulting from altered L-type calcium channel (LTCC) activity, which plays a critical role in interneuron migration: in fact, the TS migratory phenotype was rescued by application of the LTCC blocker nimodipine [54]. These results suggest the presence of dysfunctional cortical development and E/I balance in TS.

One of the latest advances in the modeling of early neurodevelopment is the use of iPSC-derived cortical organoids to model neural oscillations [83]. Synchronous oscillations are periodical and regular network activity events that emerge early in development and are dependent on balanced glutamatergic and GABAergic signaling [134]. As E/I balance is crucial for the coordination of neuronal oscillatory activity, and is one of the leading hypotheses in the etiology of ASD [25], it is not surprising that impairments in neuronal oscillatory activity and synchrony have been proposed to be correlated with social behavior deficits in ASD [135]. Thus, modeling organoid neural oscillations may allow the reproduction of a central hallmark of the in vivo brain: a highly synchronous and stereotypical network activity. Such an advancement would open new avenues in characterizing normal and dysfunctional activity in brain networks during early neurodevelopment and within ASD.

## Conclusions

The complex genetic etiologies and clinical phenotypes observed in ASD patients hinder the research of the pathophysiological mechanisms underpinning ASD and the quest for pharmacological treatments. Although animal models have deepened our understanding of disease mechanisms and shed light on possible therapeutic approaches, there has been little translational benefit for human patients. iPSC technology allows the generation of patient-specific neuronal cells, thus representing a powerful platform for investigating disease mechanisms and developing targeted therapies. Even though much work remains to be done to overcome the technical limitations of iPSC-based models (e.g., experimental variability, cellular heterogeneity, or lack of some brain cell types), their study has provided invaluable insight into the molecular and cellular mechanisms underlying ASD. In particular, a growing body of literature has provided evidence of altered balance between excitation and inhibition in human-based models of ASD. These have been linked to disruptions in different neuronal mechanisms contributing to the generation and regulation of E/I balance, such as homeostatic plasticity, synaptic transmission, and neuronal excitability. Furthermore, the recent advent of brain organoids has represented a great advance in modeling of ASD, providing a more valuable model of brain development in which to assess E/I imbalance. Indeed, organoids recapitulate several morphological and functional features of the developing human brain, and have the potential to be a more predictive drug screening platform. These advances support the study of iPSC-derived neuronal systems as key in unlocking pathogenic mechanisms and potential treatment avenues in ASD.

## Data Availability

Not applicable.

## Abbreviations

ADHD: Attention-deficit hyperactivity disorder
ASD: Autism spectrum disorder
CNV: Copy number variation
E/I: Excitation/inhibition
EEG: Electroencephalography
EPSC: Excitatory postsynaptic current
FXS: Fragile X syndrome
GABA: Gamma-aminobutyric acid
HCN: Hyperpolarization-activated cyclic nucleotide-gated
ID: Intellectual disability
iPSC: Induced pluripotent stem cell
LTCC: L-type calcium channel
KO: Knockout
MEA: Multi-electrode array
MEG: Magnetoencephalography
MRS: Magnetic resonance spectroscopy
NSC: Neural stem cell
OCD: Obsessive-compulsive disorder
PC: Purkinje cells
PMS: Phelan-McDermid syndrome
RA: Retinoic acid
RTT: Rett syndrome
TS: Timothy syndrome
TSC: Tuberous sclerosis complex
